# Autologous Diced Cartilage in Nasal Septoplasty

**Published:** 2016-09

**Authors:** Sameh Ibrahim Sersar, Ibrahim Yassin, Mohammed Saad Eldin Aly

**Affiliations:** 1 Mansoura University, Cardiothoracic Surgery Department, 35516, Mansoura, Egypt;; 2 KAMC, Makkah, Saudi Arabia;; 3 Saud AlBabtain Cardiac Centre, Dammam and Tanta University Hospital, Egypt;; 4 ENT Department, KAMC, Makkah, Saudi Arabia

**Keywords:** Autologous cartilage, Nasal septoplasty, Rectus fascia

## Abstract

Diced rib cartilage is an acceptable option in severe nasal deformities. We present our preliminary experience in KAMC in nasal septoplasties using the autologous diced costal cartilage. This is a retrospective study of the 22 cases who needed the autologous diced costal cartilage in our centre in 4 years. All our patients needed autologous diced rib cartilages. Twelve were wrapped with temporalis fascia, eight needed rectus fascia and perichondrium was used in only 2 cases. The naso-frontal angle for the whole series decreased by a mean of 4.41° (*p*=0.008) for the group using the rectus fascia diced cartilage graft. From the aesthetic point of view, all cases were satisfied except 3 (13.6%); two in the group of diced cartilage temporalis fascia; group 1. From the functional breathing view, only 1 case was not satisfied. He was in group 1. Autologous rib cartilage was shown to be a good graft in nasal septoplasty especially if wrapped with rectus fascia.

## INTRODUCTION

Rib cartilage is an important source for grafts required when significant dorsal nasal augmentation or structural support is mandated. Due to the problem of the graft wrap and twist, limited visibility and sometimes displacement, it can be wrapped in fascia. This wrapping technique of the diced cartilage has been evolved and improved over decades.^1^^-^^5^ Cartilage grafts can be whole, crushed, diced or chimeric. They can be autologous or homologous irradiated.^2^^-^^5^ Autologous cartilage graft use to reconstruct the nasal deformities was previously described.^2^^,^^3^^,^^6^^-^^13^ We aimed to review and present our preliminary experience in King Abdullah Medical City; Makkah, Saudi Arabia in the last four years in using autologous diced rib cartilage grafts in the nasal septoplasties.

## CASE REPORT

Between 2011 and 2015, we presented 22 cases of nasal septoplasties who needed autologous rib cartilage in KAMC; Makkah in the past 4 years. An institutional research board (IRB) approval from our local hospital was obtained to collect the data. A thorough history was taken to specify the etiology of the nasal deformity. Complete head and neck examination especially the palate, side walls of the nose and turbinates. The dynamic function of the nasal valves was sometimes assessed. We needed to delineate the anatomic relations of the nasal support structures with the nasal dorsum. 

Routine preoperative labs and anesthesia consultations were done. Preoperative, early postoperative and 6 months photographs were taken. The risk potentials of impaired nasal breathing, numbness of the nasal tip, change skin color and texture of the nose, decreased nasal stability were explained to the patient and family. An informed consent was signed by the competent patient of guardian. The possibility of needed revision was discussed with the patient. We used to have Basal view standard photograph with the help of Nikon D60 camera and AF-S Micro Nikkor lens (60 mm 1:2.8 g ED). The photographs were taken at 15 cm distance at the same angles to define the naso-frontal angle and nasal cross sectional area (NCSA). 

The surgical procedure was similar to the Turkish delight technique described before^8^ including harvesting the autologous rib cartilage, usually right sided 5^th^ or 8^th^ rib cartilage, cut in small pieces of 0.5 to 1 mm, then it is wrapped in one layer of Surgicel, fascia (temporalis or rectus abdominis) or perichondrium and then it was kept wet with an antibiotic. The cartilage graft was then molded into a cylinder shape to be inserted under the dorsal nasal skin. After completing the mucosal stitching, the molded plasticine like graft was packed under the dorsal skin. If the depression of the nose was mild to moderate, then either the septal and or the conchal cartilages were used to augment the nasal dorsum with an acceptable, durable and effective outcome. 

If the nasal defect was severe, then the costal cartilage was necessary to correct not only the nasal length and shape but also the columellar projection. In recurrent or postoperative cases, this technique was a suitable solution. The dorsal part of the deviated septal cartilage was over-excised and surgical, fascia or perichondrium-wrapped diced cartilage were inserted and packed till a straight nose was obtained. Autologous costal cartilage harvest was done either before, during or after preparation of the nasal septum depending on the availability of the thoracic surgeon. Right sided rib cartilages were always used. 

An incision of 3 cm was made just below the mammary crease or in the right costal margin 1 space below and 4 cm to the right of the xiphoid process to harvest the right 7^th^, 8^th^ or 9^th^ costal cartilages. Sharp and diathermic dissection follows down to the level of the rectus muscle. The fascia overlying the muscle was harvested as a square of 6×6 cm and then muscle fibers were bluntly retracted or divided to identify the costal cartilage. Then the perichondrium was incised along its axis. Using the freer, the rib cartilage was encircled and harvested with or without its perichondrium. 

Then, the muscle and fascia were repaired with a 2-0 absorbable suture. The pleura were tested by the absence of air bubbles from a pool of normal saline in the donor site. After good hemostasis, the chest wall donor site was closed in layers, including the perichondrial layer. The harvested cartilage was ground with a bone mill and kept in normal saline solution and antibiotic.  The septoplasty was performed as described before.^8^^,^^11^^,^^12^ Lidocaine with 1:100,000 epinephrine was used to infiltrate the area. A trans-columellar incision was used after local infiltration anesthesia, followed by periosteal incision and elevation up to the radix and separation of the upper lateral cartilage. The dorsal septum was resected followed by resection of the bonny hump, osteotomy and resection of the upper lateral cartilage then the rib cartilage graft was fixed between the septum and upper lateral cartilages. The lower lateral cartilages were fixed using non-absorbable 6-0 prolypropylene. 

The nasal tip profile was enhanced by using the columellar strut graft with tip grafts and tip contouring with sutures. The transculomellar incision was closed with 6-0 polypropylene and the infra-cartilagenous incisions were closed using 5-0 absorbable sutures. The non-absorbable sutures were removed on the fifth postoperative day. The septum was fixed by anchoring quilting sutures. Both nostrils were packed with antibiotic ointment containing paraffin.^7^^,^^8^^,^^10^^,^^11^ All patients except 2 were regularly followed in the outpatient clinic. They were clinically assessed and a questionnaire of aesthetic and functional breathing satisfaction were answered and kept in their files. Photographs were taken at 1 and 6 months. These patients were followed for a mean duration of 29 months with a range between 1-47 months. 

The male to female ratio was 2.66/1. Their mean age was 22 years with a (range=19-62 years). The causes of the nasal deformity were trauma (45.4%), previous surgery (45.4%), congenital (4.6%) and infection (4.6%). All patients needed autologous diced rib cartilages. Twelve were wrapped with temporalis fascia, eight needed rectus fascia and perichondrium was used in only 2 cases. The operation time was less in cases of groups (2 and 3) as the rectus fascia was in our way to the rib cartilage so, it was harvested before the rib cartilage. We did not need to have any other incision and scar in the temporal region. The naso-frontal angle for the whole series decreased by a mean of 4.41° with a *p* value of 0.008 for group 2. 

The mean nasofrontal angle (NFA) improvement was more than 3° in group 1, almost 7° in group 2 and 3.34° in group 3. There was one case of nasal irregularity in the group of diced cartilage with temporalis fascia (4.6%). These results are presented in table 1. From the aesthetic point of view, all cases were satisfied except 3 (13.6%); two in group 1 and one in group 2. From the functional breathing view, only 1 case was not satisfied (group 1). 

No complications were reported in the donor site. There was no seroma, hematoma or abscess. We never had pneumothorax, hemothorax, hypertrophic scar or keloid in our cases. We had one superficial wound infection in the recipient site which was controlled by antibiotics and dressings.

**Table 1:** Preoperative and 6 months postoperative nasofrontal angle and nasal irregularities

**Variable**	**Diced cartilage with temporalis fascia**		**Diced cartilage with rectus fascia**		**Diced cartilage with perichondrium**	
	**Pre-Op** **Group 1**	**Post-Op** **Group 1**	**Pre-Op** **Group 2**	**Post-Op Group 2**	**Pre-Op** **Group 3**	**Post-Op Group 3**
Nasofrontal angle mean improvement	143.76	140.62	146.10	139.5	144.26	140.92
Nasal irregularity	12	1	8	0	2	0

## DISCUSSION

Rhinoplasty aims at a nice nasal dorsum whose height and dorsal aesthetic lines are normal with a refined nasal tip, lengthened nose and normal or near to normal columello-labial angle and naso-frontal angle.^12^^,^^13^ Our series aimed to correct mainly the traumatic and postoperative deformities (90.8% both), and rarely congenital nasal deformities and infections (4.54% each) according to our hospital policy. Dorsal nasal grafts are the most challenging grafts due to the fact that they are placed under the rhinion which is very thin leading to obvious visible irregularities and mal contour if they were uneven, misplaced or displaced. As many things in life, the ideal is not there and it is just a dream; being a “continuing quest”. Synthetic materials were thought to give the best results but they have not been universally accepted due to the high risks of extrusion and infection. Graft warp, resorption and infection problems limited to a great extent the use of irradiated homologous rib cartilages.^12^^-^^17^

We encountered the problems of insufficient amount and or quality of available cartilage during rhinoplasty especially if there is more ethmoid and vomer than septal cartilage, so it became more difficult to carve an appropriately sized graft with an acceptable quality. In such a case, the auricular cartilage could be an acceptable option; however, its curve may not give the essential structural and functional support that an incompetent endo-nasal valve needs. In our practice, we used the costal cartilages in 22 cases out of 71 septoplasties; (31%) of our practice. So, we did not routinely use diced costal cartilages but only when needed. This goes with other series who stated that rib cartilage grafting has became a good alternative option. It is a good substitute not only for those who do not have enough/adequate septum but also because ear cartilage may not have the structural force that one may need.^7^^,^^17^^,^^18^

We had a series of 71 cases of septoplasties. We used autologous grafts as their consistency is similar to the nasal tissues, they are easily carved to the required size and shape with minimal resorption and with no manifeations of rejection.^12^^,^^13^ In one study, the 5^th^ rib on the right side was harvested despite lacking a reasonable medical evidence that is the best rib to use. It seems mostly a matter of individual choice and personal preferences to which rib is going to be selected as the donor site.^13^

In our series, we found that the use of the right 7^th^, 8^th^, or 9^th^ ribs are better as they are more easily harvested, with a more amount of costal cartilages with a good amount of rectus fascia overlying. The chance to injure the pleura here is very minimal if any. Despite the inner core calcification of the rib that may lead to a more breakable graft, it helps to avoid bending or warping of the graft. In several studies, it was proved with statistically significant superior outcomes of the costal cartilage than the septal cartilage to increase the nasal cross sectional area.^7^^-^^13^ The costal cartilage was practically the only material with sufficient strength and volume to achieve the reconstructive goals in nasal septoplasties.^13^

The means of normal males’ nasofrontal, angle are 123.85°±13.23°; while those of normal females are 133.16°±8.88°; with a range between 123.85 and 144.04°. They are measured between the glabella, nasion and pronasale believing that the average values may be used as a guide in corrective esthetic–cosmotic surgeries.^19^ All our patients had similarly a postoperative naso-frontal angle within the normal range.  The results of using the rectus fascia to wrap the diced cartilages were superior to using the temporalis fascia. Group 2 had a shorter operation time, smaller scar, larger amount of fascia, better aesthetic and functional satisfactions. These results cope with those who needed major dorsal augmentation and found that patients in the diced cartilage rectus fascia group had not only significantly shorter operating room times with significantly higher average subjective and objective satisfaction scoresbut also, they had significantly shorter postoperative hospital stays than did other patients in the diced cartilage temporalis fascia group.^1^

A meta-analysis including ten series of 491 cases of rhinoplasties using the autologous diced cartilages found a low rate of complications and morbidities. Graft wrap and chest site scar rates were relatively high; mandating the surgical team to pay utmost attention. The follow-up mean time was 33.3 months. The need to revision surgery as the commonest complication was reported in 14.07% (95% CI, 6.19%-24.20%). Graft warp rate was 3.08% (95% confidence interval [CI], 0%-10.15%), rate of resorption was 0.22% (95% CI, 0%-1.25%), graft site infection was 0.56% (95% CI, 0%-2.61%) and graft displacement was 0.39% (95% CI, 0%-1.97%). Hypertrophic chest scar was 5.45% (95% CI, 0.68%-13.24%). Pneumothorax was reported in 0% (95% CI, 0%-0.32%).^20^

One of the solutions to overcome the graft wrap is the use of chimeric autologous rib cartilage which is obtained by the combination of bone and cartilage in the graft to get a relatively stable a single dorsal onlay graft.^21^ We showed that autologous rib cartilage is a good graft in nasal septoplasty especially if wrapped with rectus fascia, even the limitations of our study were small sample size and a relatively short follow up. 

## CONFLICT OF INTEREST

The authors declare no conflict of interest.

## References

[1] As’adi K, Salehi SH, Shoar S (2014). Rib Diced Cartilage-Fascia Grafting in Dorsal Nasal Reconstruction: A randomized clinical trial of wrapping with rectus muscle fascia vs deep temporal fascia. Aesthet Surg J.

[2] Goodale JL (1901). A case of nasal deformity from a median furrow corrected by subcutaneous implantation of the septal cartilage. Boston Med Surg J.

[3] Calvert J, Brenner K (2008). Autogenous dorsal reconstruction: maximizing the utility of diced cartilage and fascia. Semin Plast Surg.

[4] Menger DJ, Nolst Trenité GJ (2010). Irradiated homologous rib grafts in nasal reconstruction. Arch Facial Plast Surg.

[5] Kridel RW, Ashoori F, Liu ES, Hart CG (2009). Long-term use and follow-up of irradiated homologous costal cartilage grafts in the nose. Arch Facial Plast Surg.

[6] Peer LA (1948). Reconstruction of the auricle with diced cartilage grafts in a Vitallium ear mold. Plast Reconstr Surg.

[7] Wulkan M1, Sá AJ, Alonso N (2012). Modified technique to increase nostril cross sectional area after using rib and septal cartilage graft over alar nasal cartilages. Acta Cir Bras.

[8] Erol OO (2000). The Turkish delight: a pliable graft for rhinoplasty. Plast Reconstr Surg.

[9] Yang HC, Cho HH, Jo SY, Jang CH, Cho YB (2015). Donor-site morbidity following minimally invasive costal cartilage harvest technique. Clin Exp Otorhinolaryngol.

[10] Mohmand MH, Ahmad M (2014). Component rhinoplasty. World J Plast Surg.

[11] Daniel RK, Calvert JW (2004). Diced cartilage grafts in rhinoplasty surgery. Plast Reconstr Surg.

[12] Daniel RK (2008). Diced cartilage grafts in rhinoplasty surgery: current techniques and applications. Plast Reconstr Surg.

[13] Isac C, Mihajlovic D, Bratu T, Isac A (2012). Severe saddle nose deformity reconstructed with rib cartilage. Chirurgia (Bucur).

[14] Hiraga Y (1980). Complications of augmentation rhinoplasty in the Japanese. Ann Plast Surg.

[15] Sheen JH (1998). The ideal dorsal graft: a continuing quest. Plast Reconstr Surg.

[16] Clark JM, Cook TA (2002). Immediate reconstruction of extruded alloplastic nasal implants with irradiated homograft costal cartilage. Laryngoscope.

[17] Kelly MH, Bulstrode NW, Waterhouse N (2007). Versatilty of diced cartilage-fascia grafts in dorsal nasal augmentation. Plast Reconstr Surg.

[18] Baser B, Kothari S, Thakur M (2013). Diced cartilage: an effective graft for post-traumatic and revision rhinoplasty. Indian J Otolaryngol Head Neck Surg.

[19] Uzun A, Ozdemir F (2014). Morphometric analysis of nasal shapes and angles in young adults. Braz J Otorhinolaryngol.

[20] Wee JH, Park MH, Oh S, Jin HR (2015). Complications associated with autologous rib cartilage use in rhinoplasty: a meta-analysis. JAMA Facial Plast Surg.

[21] Hsiao YC1, Abdelrahman M, Chang CS, Chang CJ, Yang JY, Lin CH, Chang SY, Chuang SS (2014). Chimeric autologous costal cartilage graft to prevent warping. Plast Reconstr Surg.

